# Bis‐Tridentate Iridium(III) Phosphors with Very High Photostability and Fabrication of Blue‐Emitting OLEDs

**DOI:** 10.1002/advs.201800846

**Published:** 2018-07-31

**Authors:** Hsin‐Hung Kuo, Ze‐lin Zhu, Chun‐Sing Lee, Yi‐Kuang Chen, Shih‐Hung Liu, Pi‐Tai Chou, Alex K.‐Y. Jen, Yun Chi

**Affiliations:** ^1^ Department of Chemistry and Frontier Research Center on Fundamental and Applied Sciences of Matters National Tsing Hua University Hsinchu 30013 Taiwan; ^2^ Center of Super‐Diamond and Advanced Films (COSDAF) and Department of Chemistry City University of Hong Kong Hong Kong SAR; ^3^ Department of Chemistry National Taiwan University Taipei 10617 Taiwan; ^4^ Department of Materials Science and Engineering and Department of Chemistry City University of Hong Kong Hong Kong SAR

**Keywords:** bis‐tridentate, carbazole, iridium, N‐heterocyclic carbene (NHC), pyrazolate

## Abstract

Sky‐blue and blue‐emitting, carbazolyl functionalized, bis‐tridentate Ir(III) phosphors **Cz‐1**–**Cz‐3** with bright emission and short radiative lifetime are successfully synthesized in a one‐pot manner. They exhibit very high photostability against UV–vis irradiation in degassed toluene, versus both green and true‐blue‐emitting reference compounds, i.e., *fac*‐[Ir(ppy)_3_] and *mer*‐[Ir(pmp)_3_]. Organic light‐emitting diodes (OLEDs) based on **Cz‐2** exhibit maximum external quantum efficiency (EQE) of 21.6%, EQE of 15.1% at 100 cd m^−2^, and with CIE*_x_*_,_ *_y_* coordinates of (0.17, 0.25). This study provides a conceptual solution to the exceedingly stable and efficient blue phosphor. It is promising that long lifespan blue OLED based on these emitters can be attained with further engineering of devices suitable for commercial application.

Organic light‐emitting diodes (OLEDs) are being considered as one most appealing technologies of solid‐state lighting and flat panel display applications.^1^ The use of this technology requires the employment of light‐emitting materials with three elementary colors, i.e., red, green, and blue. However, development of highly efficient and stable blue emitters remains demanding in comparison to green and red counterparts for which their lower energy gaps make them less susceptible to the formation of highly energetic, hot excited states generated by exciton–polaron and/or exciton–exciton annihilation.^2^ Although, certain pure organic thermally activated delay fluorescent (TADF) emitters have already showed satisfactory blue CIE*_x_*_,_ *_y_* chromaticity,^3^ the long‐lived operational stability has not yet been achieved.^4^ Similarly, both Pt(II)‐ and Ir(III)‐based emitters have been claimed to display blue CIE*_x_*_,_ *_y_* chromaticity,^5^ but has rather limited success in terms of exhibiting good operational lifespan.^6^

To our understanding, the efficient and stable iridium(III) metal‐based emitters, blue in particular, should possess i) both the robust and rigid framework to avoid conformational distortion upon excitation, as it would facilitate the nonradiative deactivation, ii) strong metal–ligand bonding interaction to destabilize the high‐lying metal‐centered (dd*) excited states, for which the thermal population to these states with repulsive potential energy surface would cause serious quenching of emission,^7^ iii) shortened radiative lifetime to reduce the population density of the long‐lived triplet excitons in devices, alleviating the exciton–exciton and triplet‐polaron annihilation.^8^ Based on these considerations, we proposed that the demanded rigid structure can be achieved using the so‐called bis‐tridentate (3 + 3) coordination architecture, which deems to be more stable than the *tris*‐bidentate (2 + 2 + 2) design presented in the traditional blue emitters such as FIrpic,^9^ MS2,^10^ and [Ir(fdpt)_3_].^11^ For fulfilling the second criterion, the N‐heterocyclic carbene (NHC) is employed as the preferred donor versus the N‐donor such as pyridine and azolate in assembling the demanded metal emitter, as the former is known to exert more destabilized lowest unoccupied molecular orbital (LUMO) and simultaneously has greater ligand field strength.^12^ Third, decreasing the ligand‐to‐ligand charge transfer (LLCT) and intraligand charge transfer contribution, but increasing metal‐to‐ligand charge transfer (MLCT) characters could reduce the emission radiative lifetime due to the heavy metal atom induced spin–orbit coupling.^13^ These can be achieved by strategically enriching the electron density of the central metal atom. In this communication, very stable and highly efficient blue‐emitting bis‐tridentate Ir(III) complexes are synthesized according to these three fundamental principles.

The bis‐tridentate Ir(III) complexes have been independently reported by Williams and co‐workers,^14^ De Cola and co‐workers,^15^ and Esteruelas et al.^16^ However, inferior emission efficiency and lack of color tunability were the major weaknesses encountered to these earlier researches. To circumvent these obstacles, we set forth preparation of bis‐tridentate Ir(III) complexes using both pincer dicarbene and functional 6‐pyrazolyl‐2‐phenylpyridine (pzyph) as the ancillary and chromophoric chelates, respectively.^17^ They were synthesized in a one‐pot manner, i.e., heating of a 1:1:1 mixture of IrCl_3_·3H_2_O, carbene pincer pro‐chelate, pzyph chelate, and excess of potassium acetate in propionic acid.^18^ The added metal acetate works as a cyclometalation promoter via a sequence of concerted metalation and deprotonation reaction.^19^ Representative sky‐blue and blue‐emitting Ir(III) complexes [Ir(mimf)(pzyph^F^)] (**SB**) and [Ir(mimb)(pzy^tB^Oh^F^)] (**Px‐5**) were obtained (cf. **Scheme** **1**), for which the prime difference in structure was the possession of an oxygen spacer in pzy^tB^Oh^F^ chelate of **Px‐5**. It disrupts the effective π‐conjugation and raises the ππ* energy gap versus that of the parent pzyph chelate in **SB**. Nevertheless, both Ir(III) emitters showed excellent emission efficiency, i.e., 95% in doped 9‐(3‐(9*H*‐carbazol‐9‐yl)phenyl)‐9*H*‐carbazole‐3‐carbonitrile (mCPCN) host for **SB** and 91% in doped DPEPO (bis[2‐(diphenylphosphino)phenyl]ether oxide) host for **Px‐5**, and afforded blue‐emitting OLEDs with maximum external quantum efficiency (EQE) and with CIE*_x_*_,_ *_y_* coordinates of 27.0% and (0.18, 0.40), and 20.7% and (0.15, 0.17), respectively.^18^

**Scheme 1:**
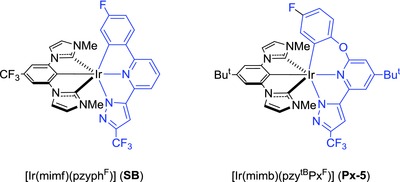
Structure of the representative bis‐tridentate Ir(III) phosphors **SB** and **Px‐5**.

Recently, we were inspired by a report on the bond dissociation energy (BDE) of organic materials for optoelectronics,^20^ which indicated that the strong C(sp^2^)—N(sp^2^) bonding interaction in carbazolyl derivatives is helpful in providing greater stability in phosphorescence organic light‐emitting diodes (PhOLEDs). Therefore, we were encouraged to employ the carbazolyl fragment as the replacement of original phenoxy group in the class of pzyph chelate. The structural drawing of [(pzy^tB^Cz^F6^)H_2_] is depicted in **Scheme** **2**, and its preparation follows a three‐step procedure. First, coupling of 2‐bromo‐4‐*tert*‐butyl‐6‐acetylpyridine and 3,6‐bis(trifluoromethyl)‐9*H*‐carbazole^21^ afforded 9‐(4‐*tert*‐butyl‐6‐acetylpyridin‐2‐yl)‐3,6‐bis‐(trifluoromethyl)‐9*H*‐carbazole. It was next subject to Claisen condensation with ethyl trifluoroacetate, followed by hydrazine cyclization to yield tridentate chelate [(pzy^tB^Cz^F6^)H_2_]. The schematic reaction diagram, employed chemicals, and detailed conditions are depicted in the Supporting Information. Additionally, three dicarbene pincer ancillaries, i.e., [(mimb)H_3_·(PF_6_)_2_], [(mimf)H_3_·(PF_6_)_2_], and [(pimf)H_3_·(PF_6_)_2_], each with methyl (m) or isopropyl (p) substituent on imidazolium (im) fragment and *tert*‐butyl (b) or trifluoromethyl (f) substituent at the central phenyl group, were selected for tuning both the steric and electronic properties. Next, the Ir(III) complexes **Cz‐1**–**Cz‐3** (**Scheme** **3**) were obtained by heating a mixture of both chelates, IrCl_3_·3H_2_O and K_2_CO_3_ in propionic acid. The single crystal X‐ray diffraction study on a parent carbazolyl complex [Ir(mimb)(pzy^tB^Cz)] (**Cz‐0**) was also conducted to confirm the structures, cf. data depicted in ESI. Scheme 3 depicted the structural drawings of these Ir(III) metal complexes **Cz‐1**–**Cz‐3**. Importantly, we have selected dual CF_3_ substituents instead of fluoro groups in functionalizing the carbazolyl appendage of Ir(III) complexes **Cz‐1**–**Cz‐3**, for avoiding the known defluorination reaction of C(aryl)‐F entity.^22^

**Scheme 2:**
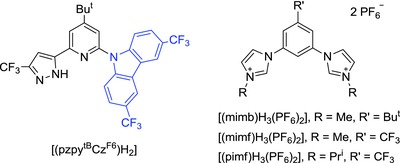
Drawings of the studied chromophoric chelates (left) dicarbene pincer (right).

**Scheme 3:**
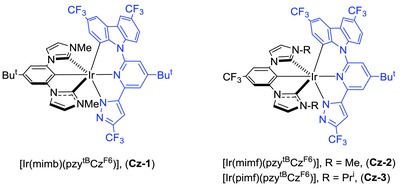
Molecular structure of the studied Ir(III) phosphors **Cz‐1**–**Cz‐3**.

UV–vis absorption and emission spectra were recorded in CH_2_Cl_2_ solution and the data were provided in **Figure** **1** and **Table** **1**, respectively. The intense absorption above 330 nm rooted from ligand‐centered ππ* transition, while the absorption at the longer wavelength region 370–450 nm stemmed from both MLCT transitions in singlet and triplet manifolds, respectively. The structureless emission profile of complexes **Cz‐1**–**Cz‐3** was recorded with peak max. at 486, 473, and 476 nm, and nearly unitary photoluminescence quantum yield (PLQY,*Φ*) was observed for **Cz‐1**, and turned slightly low to ≈83% for both **Cz‐2** and **Cz‐3** in degassed CH_2_Cl_2_ at room temperature (RT). Surprisingly, the radiative lifetime (τ_rad_) of emitters **Cz‐1**–**Cz‐3** (2.77–3.80 µs) was sufficiently shorter than the previously reported Ir(III) reference complexes **SB** and **Px‐5** (τ_rad_ = 5.41 and 12.0 µs),^17^, ^18^ manifesting the dominant charge transfer (i.e., both MLCT and LLCT) contribution in these carbazolyl coordinated Ir(III) complexes.

**Figure 1:**
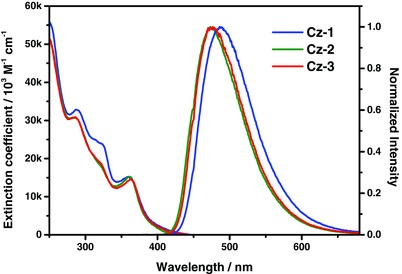
UV–vis absorption and normalized emission spectra of Ir(III) complexes **Cz‐1**–**Cz‐3** recorded in CH_2_Cl_2_ solution at RT.

**Table 1:** Essential photophysical and relevant data of Ir(III) complexes **Cz‐1**–**Cz‐3**

	Abs. λ_max_ [nm] (ε × 10^3^)a)	Em λ_max_ [nm]a)	*Φ* [%]a), b)	*Φ* [%]c)	τ_obs_ [µs]a)	τ_r_ [µs]a)	FWHMd)	*E* ^ox^_½_ (Δ*E*_p_) [V]e)	*E* ^re^_pc_ [V]e)	*T*_d_ [°C]f)
**Cz‐1**	289(33), 324.5(24), 358(15)	486	100	80.3	2.77	2.77	3420	0.55 (0.09)	−2.98	423
**Cz‐2**	287(31), 361(15)	473	83.5	86.9	3.20	3.83	3440	0.69 (0.08)	−2.81	448
**Cz‐3**	287(31), 322(19), 363.5(15)	476	82.7	95.6	2.7	3.26	3400	0.68 (0.09)	−2.79	386

^a)^ All photophysical data were recorded in CH_2_Cl_2_ with a conc. of 10^−5^_M_ at RT, ε is in _M_^−1^ cm^−1^

^b)^ Emission was measured with excitation at 375 nm and in reference to Coumarin 102 in MeOH (*Φ* = 80%)

^c)^ Measured in doped film (10 wt% in DPEPO matrix)

^d)^ Full width at half‐maxima of PL in cm^−1^

^e)^ *E*_½_ = [(*E*_pa_ + *E*_pc_)/2] and Δ*E*_p_ = |*E*_pa_ − *E*_pc_| in V, while *E*_pa_ and *E*_pc_ are defined as the anodic and cathodic peak potentials referenced to Fc^+^/Fc

^f)^ *T*_d_ is the temperature with 5% of weight loss in thermogravimetric analysis.

Also conducted was the computational approach based on time‐dependent density functional theory (TD‐DFT) at the B3LYP/LANL2DZ (Ir) and B3LYP/6‐31g(d,p) (H, C, N, F, Cl) levels using CH_2_Cl_2_ as the solvent (see Supporting Information for detail). The calculated energy in terms of wavelengths and assignments of each electronic transition of Ir(III) complexes **Cz‐1**–**Cz‐3** are listed in **Table** **2** and Tables S1–S3 in the Supporting Information. **Figure** **2** and Figures S2–S4 in the Supporting Information depict the frontier orbitals involved in the lower‐lying transitions. The calculated wavelengths of the S_0_ → S_1_ optical transition for **Cz‐1**: 385 nm, **Cz‐2**: 377 nm, and **Cz‐3**: 379 nm were close to the observed onsets of the experimental absorption peaks depicted in Figure 1. The calculated S_0_ → T_1_ transition wavelengths for **Cz‐1**: 421 nm, **Cz‐2**: 417 nm, and **Cz‐3**: 418 nm were also in good agreement with the trend of the onset of their phosphorescence spectra in Figure 1. Moreover, after geometry optimization, the calculated wavelengths of the T_1_ → S_0_ emission transitions were at 527, 513, and 510 nm for **Cz‐1**, **Cz‐2**, and **Cz‐3**, respectively. These values are ≈4–4.5 kcal mol^−1^ lower in energy than that of the experimentally acquired emission peak wavelengths (see Figure 1), which is common if one considers that the computational approach is subject to few kcal mol^−1^ uncertainty, depending on the theoretical levels and basis sets being applied. Nevertheless, the trend of energy gap obtained by the theoretical approach is consistent with that of the experimental result. Therefore, the current TD‐DFT simulation works well in predicting the lowest Franck–Condon transition for both absorption and emission based on the optimized ground state (S_0_) structure for the studied Ir(III) complexes.

**Table 2:** The calculated wavelengths, transition probabilities and main charge characters of the lowest optical transitions S_1_ and T_1_ for Ir(III) complexes **Cz‐1**–**Cz‐3** in CH_2_Cl_2_ solution

Complex	State	λ [nm]	*f*	Main assignments	MLCT
**Cz‐1**	T_1_	421.7	0	HOMO → LUMO (43%)	21.60%
	S_1_	384.9	0.0405	HOMO → LUMO (97%)	29.94%
**Cz‐2**	T_1_	417.5	0	HOMO → LUMO (33%)	22.38%
	S_1_	377.4	0.0552	HOMO → LUMO (98%)	28.09%
**Cz‐3**	T_1_	418.5	0	HOMO → LUMO (34%)	22.29%
	S_1_	379.4	0.0497	HOMO → LUMO (98%)	28.77%

**Figure 2:**
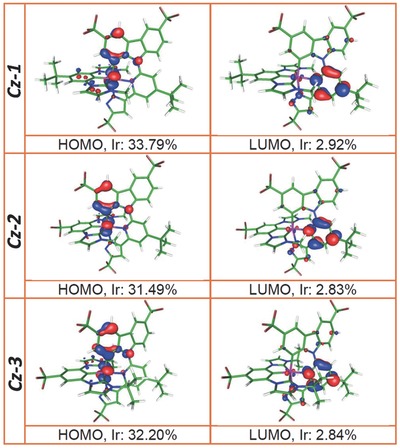
Frontier molecular orbitals for the ground state S_0_ of Ir(III) complexes **Cz‐1**–**Cz‐3** in CH_2_Cl_2_. “Ir” indicates the relative electron density distribution at the iridium atom.

According to the calculation (see Figure 2, Figures S2–S4, Supporting Information and Table 2 and Tables S1–S3, Supporting Information), both S_0_ → S_1_ (absorption) and T_1_ → S_0_ (emission) transition for **Cz‐1**–**Cz‐3** mainly involves highest occupied molecular orbital (HOMO) and LUMO, in which the electron density distributions of HOMO are mainly localized at the central Ir atom (25–33%) and carbazolyl fragment, while LUMO are mostly distributed at the central pyridine of 2‐carbazolyl‐6‐pyrazolylpyridine fragment and very few at the central Ir(III) atom (1–4%). Since we are focusing on the emission property, for more detail, the T_1_ → S_0_ transition for **Cz‐1** is mainly assigned to LUMO → HOMO (76%) with minor LUMO → HOMO‐2 (13%). For complex **Cz‐2**, the T_1_ → S_0_ transition is assigned to mainly LUMO → HOMO (65%) with minor LUMO → HOMO‐1 (12%) and LUMO → HOMO‐3 (11%) contribution. For complex **Cz‐3**, the T_1_ → S_0_ emissive transition is assigned to mainly LUMO → HOMO (79%) and minor LUMO → HOMO‐1 (9%). Note that HOMO‐1 (**Cz‐2** and **Cz‐3**), HOMO‐2 (**Cz‐1**), and HOMO‐3 (**Cz‐2**) for **Cz‐1**–**Cz‐3** are mainly located at Ir atom, 3‐methylimidazolium and pyrazolyl fragments. Therefore, the T_1_ → S_0_ transitions for **Cz‐1**–**Cz‐3** are assigned to MLCT (20–26%) mixed with LLCT. The significant MLCT percentage (>20%) implies fast S_1_ → T_1_ (or *T_m_*, *m* > 1) intersystem crossing, resulting solely in the phosphorescence. This also supports the deduced fast radiative decay rate constant for **Cz‐1**–**Cz‐3** (vide supra, Table 1) due to the enhancement of spin–orbit coupling.

To confirm this theoretical interpretation, cyclic voltammetry was conducted and showed the reversible oxidation at the metal center and irreversible reduction at the coordinated chelate, for which the graphic and numerical data are depicted in Figure S5 in the Supporting Information and Table 1. The anodic shifts of all electrochemical potentials were observed for **Cz‐2** and **Cz‐3** versus that of **Cz‐1**, which are attributed to the electron withdrawing property of CF_3_ group in **Cz‐2** and **Cz‐3** versus that of electron donating *tert*‐butyl **Cz‐1** group in on the carbene pincer chelate.

As for the stability, thermal gravimetric analysis data illustrated in Table 1 showed *T*_d_ of **Cz‐1**–**Cz‐3** exceeding 380 °C, which are common to most Ir(III) emitters documented in literature. However, the hot excited states generated by triplet–triplet annihilation of blue emitters possess an energy of ≈6 eV,^23^ meaning that these *T*_d_ concepts are not suitable in estimating the material stability of blue emitters. To cope with this deficiency and provide better assessment on material stability, there are a number of literature reports on the use of either 400 nm LED^24^ or UV irradiation^25^ to test photostability of OLED emitters in solution and in a solid organic matrix. The photostabilities studies of bis‐tridentate Ir(III) complexes remain rare in comparison to the corresponding *tris*‐bidentate phosphors. Within this context, we attempted similar photodegradation experiment for **Cz‐1**–**Cz‐3** in deaerated toluene using the standard Atlas Suntest CPS+ Xenon Test Instrument. **Figure** **3** provides the plot of ln(*A*_t_/*A*_0_) versus irradiation time of Ir(III) emitters **Cz‐1**–**Cz‐3**. For a fair comparison, the relevant data for [*fac*‐Ir(ppy)_3_]^26^ and [*mer*‐Ir(pmp)_3_]^27^ that are known to be the best green and true‐blue emitters, are also shown in Figure 3. From this diagram, the rate constant of photodegradation was estimated to be 1.9, 2.8, and 2.5 × 10^−3^ h^−1^ for **Cz‐1**, **Cz‐2**, and **Cz‐3**, and 2.6 and 12 × 10^−3^ h^−1^ for [*fac*‐Ir(ppy)_3_] and [*mer*‐Ir(pmp)_3_], using the integrated first‐order rate law:

(1) $$\ln\left[\frac{A_{t}}{A_{0}}\right]=-kt$$

**Figure 3:**
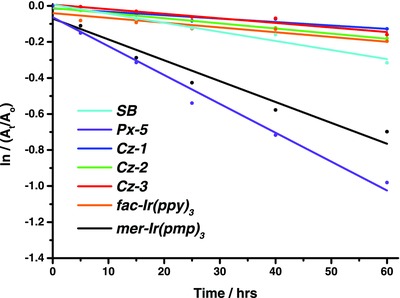
Photodegradation responses of Ir(III) emitters **SB, Px‐5, Cz‐1**–**Cz‐3**, [*fac*‐Ir(ppy)_3_], and [*mer*‐Ir(pmp)_3_], for which all samples were dissolved in deaerated toluene, placed in argon‐filled PL cuvette, followed by the simulated solar irradiation at 620 W m^−2^ and 35 °C.

These rate constants clearly verified the superior photostability of the bis‐tridentate emitters **Cz‐1**–**Cz‐3**, for which that data are comparable to that of the green‐emitting [*fac*‐Ir(ppy)_3_], but much superior to that of the true‐blue‐emitting [*mer*‐Ir(pmp)_3_] under the identical condition examined. These experimental results also corroborate the theoretical studies showing that 3 + 3 iridium skeleton can afford higher metal–ligand BDE than that of the 2 + 2 + 2 system.^23^ Moreover, the test also shows that the rate of photodegradation is also slower than that of **SB** and **Px‐5** possessing bis*‐*tridentate ligands (cf. 5.0 and 16 × 10^−3^ h^−1^). We thus propose that the carbazole moiety of the (pzy^tB^Cz^F6^) ligand forms a six‐membered nitrogen‐containing metallacycle in **Cz‐1**–**Cz‐3**, which is more stable than that of the corresponding metallacycle made by the phenylpyridine of pzyph for **SB** and the phenoxypyridine of pzyOh for **Px‐5** due to both the higher ligand field strength and robustly chelating framework.

In view of the remarkable photophysical properties of **Cz‐1**, **Cz‐2**, and **Cz‐3**, these emitters were next co‐deposited in DPEPO (*T*_1_ = 3.3 eV) matrix to evaluate their solid‐state luminescence properties.^28^ In the thin film states (10 wt% in DPEPO), PLQYs of 80.3%, 86.9%, and 95.6% and transition PL decays of 2.95, 3.80, and 3.07 µs were observed for **Cz‐1**, **Cz‐2**, and **Cz‐3**, respectively (cf. Figure S7, Supporting Information). Accordingly, OLEDs with configuration of ITO/MoO_3_ (1 nm)/TCTA (30 nm)/mCP (10 nm)/DPEPO: Ir(III) emitters at 10 wt% (35 nm)/3TPYMB (50 nm)/LiF (1 nm)/Al (120 nm) were fabricated to assess their electroluminescence performances. Herein, both TCTA (4,4′,4″‐ *tris*(carbazol‐9‐yl)‐triphenylamine) and mCP (1,3‐di(9*H*‐carbazol‐9‐yl)benzene) were served as the hole transporting materials and 3TPYMB (*tris*(2,4,6‐trimethyl‐3‐(pyridin‐3‐yl)phenyl)borane) as the electron transporting layer. As shown in **Figure** **4** and **Table** **3**, device **Cz‐1** displays sky‐blue emission with peak efficiencies (EQE: 19.6%; CE: 39.5 cd A^−1^; PE: 37.6 lm W^−1^; Commission Internationale de l'éclairage (CIE) coordinates of (0.19, 0.34)), while the **Cz‐2**‐ and **Cz‐3**‐based devices exhibited the blue emission and impressive performances, with max. EQE of 21.6% and 19.6%, CE of 31.7 and 33.8 cd A^−1^, PE of 29.3 and 31.2 lm W^−1^, and CIE*_x_*_,_ *_y_* coordinates of (0.17, 0.25) and (0.17, 0.26) respectively. These characteristics are clearly superior to those displayed by other relevant bis‐tridentate Ir(III) emitters^18^, ^29^ and fall in the ranking column of typical *tris*‐bidentate blue‐emitting Ir(III) phosphors.^30^ The recorded CIE coordinates are still deviated from the NTSC blue standard (0.14, 0.08), e.g. National Television System Committee USA. However, this shortcoming can be fixed by employment of microcavity effect and/or using color filter in commercial OLEDs.

**Figure 4:**
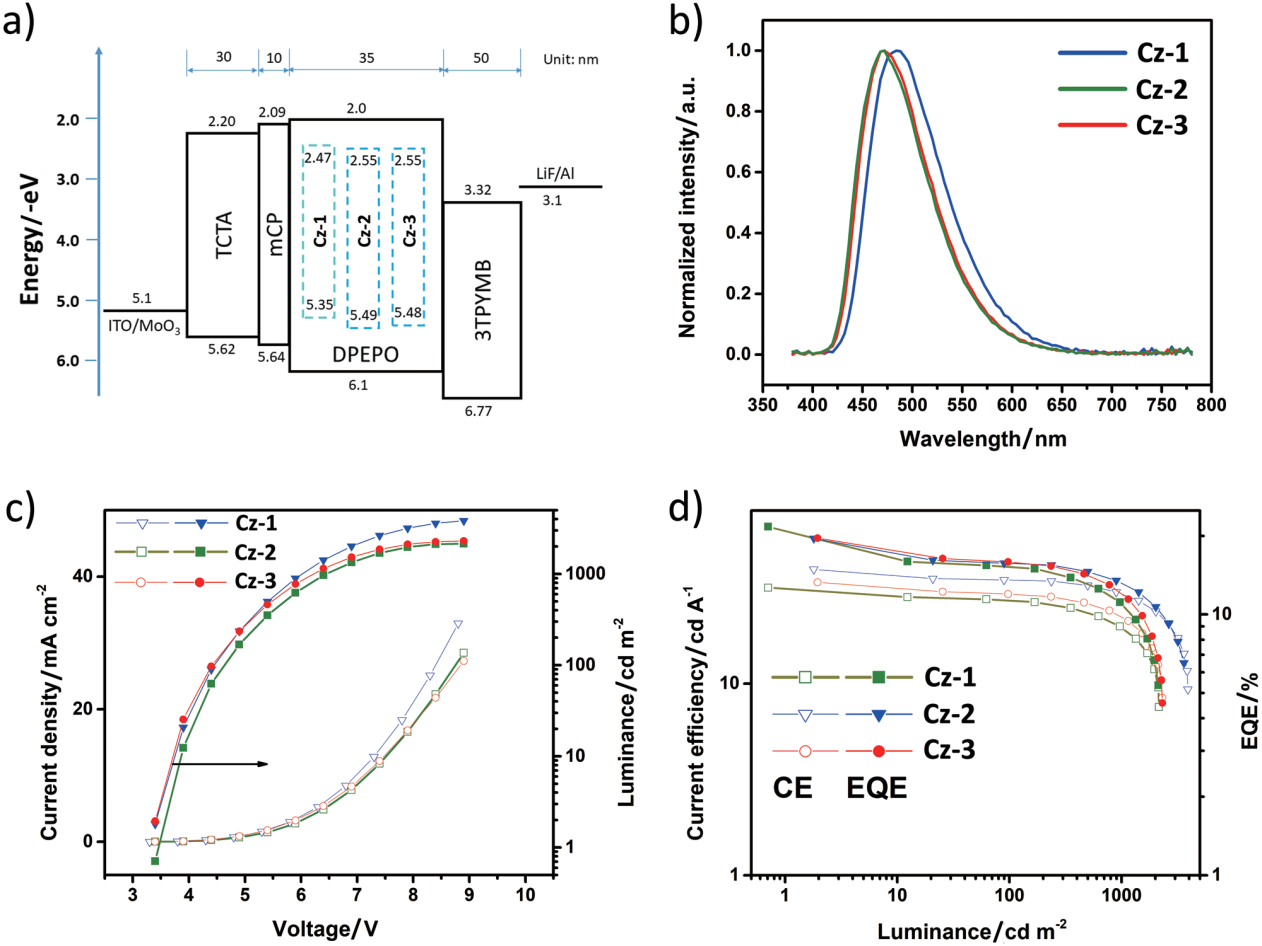
Uniformly doped blue‐emitting OLEDs: a) device diagram and energy levels of used materials, b) EL spectra of devices, c) current density–voltage–luminescence characteristics, and d) plots of current efficiency–luminance–EQE.

**Table 3:** Key performance data for the OLED devices

Emitter	Device types	*V*_on_ [V]	λ_EL_ [nm]	CEa) [cd A^−1^]	PEa) [lm W^−1^]	EQEa) [%]	CIE*_x_*_,_ *_y_* ^b)^
**Cz‐1**	Uniform	3.3	484	39.5, 34.7, 29.1	37.6, 24.3, 15.1	19.6, 15.5, 12.9	0.19, 0.34
	Graded	3.3	484	41.3, 36.2, 32.0	39.3, 24.3, 16.0	21.5, 16.5, 14.2	0.19, 0.34
**Cz‐2**	Uniform	3.5	468	31.7, 27.1, 19.9	29.3, 17.8, 9.80	21.6, 15.1, 11.0	0.17, 0.25
	Graded	3.5	468	32.5, 30.8, 24.0	29.1, 20.3, 11.6	21.0, 17.1, 13.2	0.17, 0.25
**Cz‐3**	Uniform	3.4	472	33.8, 29.4, 21.5	31.2, 21.0, 10.6	19.6, 15.9, 11.7	0.17, 0.26
	Graded	3.5	472	32.7, 32.1, 25.8	27.6, 21.3, 12.5	19.5, 17.7, 14.0	0.17, 0.26

^a)^ Data at max. and @ 100 and 1000 cd m^−2^, respectively

^b)^ @ 100 cd m^−2^. Device structure: uniformly doped: ITO/MoO_3_ (1 nm)/TCTA (30 nm)/mCP (10 nm)/DPEPO: 10 wt% dopant (35 nm)/3TPYMB (50 nm)/LiF (1 nm)/Al (120 nm); graded doping: ITO/MoO_3_ (1 nm)/TCTA (30 nm)/mCP (10 nm)/DPEPO: 15–6 wt% dopant (40 nm)/ 3TPYMB (50 nm)/LiF (1 nm)/Al (120 nm).

However, serious efficiency roll‐off was obtained as increasing luminance (Figure 4 and Table 3), especially at above 1000 cd m^−2^, which seems contradicting the better photophysical robustness of emitters. However, it should be noted that the chemical stability is not the solely factor in determining the OLED stability and efficiency roll‐off. Other device parameters such as the exciton interaction, exciton–polaron interaction, field‐induced quenching, and charge carriers imbalance also need to be considered.^31^ In an effort to unveil this efficiency roll‐off, a 2 nm thick, red‐emitting [Ir(piq)_2_(acac)] dopant was co‐deposited to the emitting layer, at a conc. of 2 wt% but with variable distances (*x* = 10, 15, and 20 nm) from the emitting layer (EML)/mCP interface.^32^ As shown in Figure S8 in the Supporting Information, the electroluminescence decreases with increase of *x*, indicating the close association of the EML/mCP interface and recombination zone (RZ). Furthermore, the red luminescence of device with *x* = 10 nm turned lowered upon increasing current and driving voltage, meaning that the RZ is further shifting closer to the EML/mCP interface.[[qv: 4c,32,33]] Therefore, in this case, the poor carrier balance in EML has resulted in a narrowed RZ and with highly concentrated excitons, causing the inferior efficiency roll‐off. This problem can be partially alleviated by adapting gradient‐doping technique,^27^ as shown in Figure S9 in the Supporting Information. For completely resolving this undesirable issue, bipolar host material with *T*_1_ > 3.00 eV^34^ is needed to prevent the possible leakage of excitons into the adjacent mCP layer, which has a triplet energy of 2.9 eV and is known to contribute considerably to the efficiency roll‐off observed in many TADF‐based true‐blue‐emitting OLEDs.[[qv: 3b,35]]

In conclusion, stability remains to be one key issue for the development of robust and efficient blue‐emitting OLEDs, for which both the material‐related and the extrinsic fabrication issues were considered to be two main factors that gave the undesirable fast device degradation. The high photostability displayed by these new bis‐tridentate Ir(III) emitters **Cz‐1**–**Cz‐3** mainly eliminates the material‐related factor. This, together with the decent OLED architecture to optimize and/or balance the carrier transport and stability of hole and electron transporting materials, would eventually provide a solution aimed at the long lifespan blue‐emitting OLED phosphors for commercial applications.

## Conflict of Interest

The authors declare no conflict of interest.

## Supporting information

Supplementary
