# Supplementary material for: Bis‐Tridentate Iridium(III) Phosphors with Very High Photostability and Fabrication of Blue‐Emitting OLEDs
Source: Adv Sci (Weinh). 2018 Jul 31;5(9):1800846. doi: 10.1002/advs.201800846 (PMC6145234; doi:10.1002/advs.201800846)
Supplement: Supplementary file 1 — Supplementary [file ADVS-5-1800846-s001.pdf]

Copyright WILEY-VCH Verlag GmbH & Co. KGaA, 69469 Weinheim, Germany, 2018.

ADVANCED SCIENCE
Open Access

Supporting Information

for *Adv. Sci.,* DOI: 10.1002/advs.201800846

Bis-Tridentate Iridium(III) Phosphors with Very High Photostability and Fabrication of Blue-Emitting OLEDs

Hsin-Hung Kuo, Ze-lin Zhu, Chun-Sing Lee, Yi-Kuang Chen, Shih-Hung Liu, Pi-Tai Chou,* Alex K.-Y. Jen,* and Yun Chi**

Copyright WILEY-VCH Verlag GmbH & Co. KGaA, 69469 Weinheim, Germany, 2016.

Supporting Information

Hsin-Hung Kuo, Ze-lin Zhu, Chun-Sing Lee, Yi-Kuang Chen, Shih-Hung Liu, Pi-Tai Chou,* Alex K.-Y. Jen,* and Yun Chi**

Hsin-Hung Kuo and Ze-lin Zhu contribute equally to this work.

Hsin-Hung Kuo, Yi-Kuang Chen, Prof. Yun Chi

Department of Chemistry and Frontier Research Center on Fundamental and Applied Sciences of Matters, National Tsing Hua University, Hsinchu 30013, Taiwan.

Email: ychi@mx.nthu.edu.tw

Ze-lin Zhu, Prof. Chun-Sing Lee

Center of Super-Diamond and Advanced Films (COSDAF) and Department of Chemistry, City University of Hong Kong, Hong Kong SAR

Email: C.S.Lee@cityu.edu.hk

Dr. Shih-Hung Liu, Prof. Pi-Tai Chou

Department of Chemistry, National Taiwan University, Taipei 10617, Taiwan.

Email: chop@ntu.edu.tw

Prof. Alex K.-Y. Jen, Prof. Yun Chi

Department of Materials Science and Engineering and Department of Chemistry, City University of Hong Kong, Hong Kong SAR.

Email: alexjen@cityu.edu.hk and yunchi@cityu.edu.hk

Experimental section:

General Information and Materials. All reactions were carried out under a nitrogen atmosphere and the solvents were dried prior to use. Commercially available chemicals were used without purification unless otherwise stated. The functional bis(imidazolium) benzene derivatives, i.e. di-hexafluorophosphate salts of 1,3-bis(3-methylimidazolium-1-yl)-5-*tert*-butylbenzene [(mimb)H_3·$(PF_6)_2$], 1,3-bis(3-methylimidazolium-1-yl)-5-(trifluoromethyl)benzene [(mimf)H_3·$(PF_6)_2$] and 1,3-bis(3-isopropyllimidazolium-1-yl)-5-(trifluoromethyl)benzene [(pimf)H_3·$(PF_6)_2$] were synthesized from the respective 1,3-dibromo-5-*tert*-butylbenzene (or 1,3-dibromo-5-(trifluoromethyl)benzene), imidazole and methyl iodide (or isopropyl iodide), followed by anion metatheses according to literature method,[1] while the dianionic chromophoric chelate 9-(4-(*tert*-butyl)-6-(3-(trifluoromethyl)-1H-pyrazol-5-yl)pyridin-2-yl)-3,6-bis(trifluoromethyl)-9H-carbazole [(pzy^{tB}Cz^{F6})H_2], were obtained using C-N coupling of 2-bromo-4-*tert*-butyl-6-acetylpyridine and 3,6-bis(trifluoromethyl)-9H-carbazole,[2] followed by Claisen condensation with ethyl trifluoroacetate, and hydrazine cyclization (**Scheme S1**).[3] ^{1}H and ^{19}F NMR spectra were measured with a Varian Mercury-400 instrument. The elemental analysis was carried out on a Heraeus CHN-O Rapid Elementary Analyzer. Field desorption (FD) mass spectra were recorded on a JEOL JMS-T200GC AccuTOF GCx instrument. The photo-induced decomposition was performed using Atlas Suntest CPS+ Xenon Test instrument.

Complexes **Cz-1** - **Cz-3** were synthesized using one-pot synthetic method: A mixture of [(mimb)H_3·$(PF_6)_2$] (662 mg, 1.13mmol), [(pzy^{tB}Cz^{F6})H_2] (647 mg, 1.13mmol), $IrCl_3$·$3H_2O$ (400 mg, 1.13 mmol) and K_2CO_3 (2.35 g, 22.6 mmol) were dissolved in propionic acid (120 mL) and heated to reflux under nitrogen for 12 hours.

After removal of solvent *in vacuo*, the remaining solid was extracted into ethyl acetate (150 mL), and the solution was washed with water (30 mL x 3), dried over anhydrous Na_2SO_4 and evaporated to dryness. Next, the obtained residue was purified by column chromatography eluting with a mixture of hexanes: ethyl acetate (2:1 to 1:1) to give a light-yellow powder of **Cz-1** (388 mg, 0.37 mmol, 32%). Other Ir(III) metal complexes **Cz-2**, **Cz-3** and **Cz-0** were synthesized in 27%, 22% and 36% of yields under similar conditions.

Selected spectral data of **Cz-1**: ^{1}H NMR (400 MHz, acetone-d$_6$, 298K, δ): 8.58 (s, 1 H, Ar H), 8.43 (d, *J* = 8.6 Hz, 1 H, Ar H), 8.15 (s, 1 H, Ar H), 8.12(s, 1 H, Ar H), 7.93 (d, *J* = 1.8 Hz, 2 H, Ar H), 7.87 (d, *J* = 8.6 Hz, 1 H, Ar H), 7.77 (s, 1 H, Ar H), 7.55 (s, 2 H, Ar H), 7.21 (s, 1 H, Ar H), 6.91 (d, *J* = 1.8 Hz, 2 H, Ar H), 6.12 (s, 1 H, Ar H), 2.98 (s, 6 H; CH_3), 1.58 (s, 9 H; CH_3), 1.52 (s, 9 H; CH_3). ^{19}F NMR (376 MHz, acetone-d$_6$, 298K, δ): -59.9 (s, 3 F; CF_3), -61.4 (s, 3 F; CF_3), -61.5 (s, 3 F; CF_3). FD MS: *m/z* (M^+) calcd for $C_{45}H_{38}F_9IrN_8$, 1054.04; found, 1054.3. Anal. Calcd. for $C_{45}H_{38}F_9IrN_8$: C, 51.28, H, 3.63, N, 10.63; Found: C, 51.19; H, 4.02; N, 10.47.

Selected spectral data of **Cz-2**: ^{1}H NMR (400 MHz, acetone-d$_6$, 298K, δ): 8.61 (s, 1 H, Ar H), 8.46 (d, *J* = 8.8 Hz, 1 H, Ar H), 8.20 (d, *J* = 1.6 Hz, 1 H, Ar H), 8.15 (d, *J* = 1.6 Hz,1 H, Ar H), 8.09 (d, *J* = 2.1 Hz, 2 H, Ar H), 7.89 (dd, *J* = 8.8 Hz, *J* = 1.8 Hz, 1 H, Ar H), 7.85 (s, 1H, Ar H), 7.78 (s, 2 H, Ar H), 7.21 (s, 1 H, Ar H), 7.00 (d, *J* = 2.1 Hz, 2 H, Ar H), 6.09 (d, *J* = 1.8 Hz, 1 H, Ar H), 3.03 (s, 6 H; CH_3), 1.59 (s, 9 H; CH_3). ^{19}F NMR (376 MHz, acetone-d$_6$, 298K, δ): -60.1 (s, 3 F; CF_3), -60.6 (s, 3 F; CF_3), -61,4 (s, 3 F; CF_3), -61.5 (s, 3 F; CF_3). FD MS: *m/z* (M^+) calcd for $C_{42}H_{29}F_{12}IrN_8$, 1065.93; found, 1066.2. Anal. Calcd. for $C_{42}H_{29}F_{12}IrN_8$: C, 47.32, H, 2.74, N, 10.51; Found: C, 47.25; H, 2.95; N, 10.73.

Selected spectral data of **Cz-3**: ^{1}H NMR (400 MHz, acetone-d_6, 298K, δ): 8.62 (s, 1 H, Ar H), 8.48 (d, *J* = 8.8 Hz, 1 H, Ar H), 8.22 (d, *J* = 1.6 Hz, 1 H, Ar H), 8.17 (d, *J* = 1.6 Hz, 1 H, Ar H), 8.16 (d, *J* = 2.1 Hz, 2 H, Ar H), 7.89 (dd, *J* = 8.8 Hz, *J* = 1.6 Hz, 1 H, Ar H), 7.85 (s, 1H, Ar H), 7.80 (s, 2H, Ar H), 7.23 (d, *J* = 2.1 Hz, 2 H, Ar H), 7.21 (s, 1H, Ar H), 6.13 (d, *J* = 1.6 Hz, 1H, Ar H), 3.65 (m, 2H; CH), 1.62(s, 9 H; CH_3), 0.87(d, *J* = 3.4 Hz, 6H; CH_3), 0.61 (d, *J* = 3.4 Hz, 6H; CH_3). ^{19}F NMR (376 MHz, acetone-d_6, 298K, δ): -60.1 (s, 3 F; CF_3), -60.6 (s, 3 F; CF_3), -61,4 (s, 3 F; CF_3), -61.5 (s, 3 F; CF_3). FD MS: *m/z* (M^+) calcd for $C_{46}H_{37}F_{12}IrN_8$, 1122.04; found, 1122.4. Anal. Calcd. for $C_{46}H_{37}F_{12}IrN_8$: C, 49.24, H, 3.32, N, 9.99; Found: C,49.34, H,3.21, N,10.05.

Selected spectral data of **Cz-0**: ^{1}H NMR (400 MHz, acetone-d_6, 298K, δ): 8.18 (d, *J* = 7.4 Hz, 1 H, Ar H), 8.08 (d, *J* = 1.4 Hz, 1 H, Ar H), 8.01 (d, *J* = 7.4 Hz,1 H, Ar H), 7.98 (d, *J* = 1.4 Hz, 1 H, Ar H), 7.91 (d, *J* = 1.9 Hz, 2 H, Ar H), 7.51 (s, 2H, Ar H), 7.47 (t, *J* = 7.4 Hz, 1 H, Ar H), 7.32 (t, *J* = 7.4 Hz, 1 H, Ar H), 7.23 (d, *J* = 7.4 Hz, 1 H, Ar H), 7.15 (s, 1 H, Ar H), 6.90 (d, *J* = 1.9 Hz, 2 H, Ar H), 6.34 (t, *J* = 7.4 Hz, 1 H, Ar H), 5.83 (d, *J* = 7.4 Hz, 1 H, Ar H), 2.99 (s, 6 H; CH_3), 1.56 (s, 9 H; CH_3), 1.52 (s, 9 H; CH_3), ^{19}F NMR (376 MHz, acetone-d_6, 298K, δ): -60.0 (s, 3 F; CF_3), FD MS : *m/z* (M^+), calcd for $C_{43}H_{40}F_3IrN_8$, 918.04 found, 918.3 Anal. Calcd. for $C_{43}H_{40}F_3IrN_8$: C, 56.26, H, 4.39, N, 12.21; Found : C, 56.51, H, 4.60, N, 12.43.

Selected crystal data of **Cz-0**: $C_{49}H_{52}F_3IrN_8O_2$; M = 1034.18; monoclinic; space group = $P2_1/c$; a = 17.7674(3) Å, b = 17.9067(3) Å, c = 15.3920(3) Å; β = 111.6194(6)°; V = 4552.55(14) Å^3; Z = 4; $\rho_{Calcd.}$ = 1.509 Mg·m^{-3}; F(000) = 2088; crystal size =0.144 x 0.075 x 0.056 mm^3; λ(Mo-K_α) = 0.71073 Å; T = 150(2) K; μ = 2.994 mm^{-1}; 29282 reflections collected, 10378 independent reflections (R_{int} = 0.0383), GOF = 1.049, final $R_1[I > 2\sigma(I)]$ = 0.0274 and wR_2(all data) = 0.0510. The

supplementary crystallographic data of **Cz-0**, CCDC 1840911, can be obtained free of charge from the Cambridge Crystallographic Data Center via www.ccdc.cam.ac.uk/data_request/cif.

Electrochemistry: Cyclic voltammetry was conducted on a CHI621A Electrochemical Analyzer. Ag/Ag^+ electrode was employed as reference electrode. The oxidation and reduction potentials were measured using a platinum working electrode with 0.1 M of NBu_4PF_6 in CH_2Cl_2 and a gold wire with 0.1 M of NBu_4PF_6 in THF, respectively. The potentials were referenced externally to the ferrocenium/ferrocene (Fc^+/Fc) couple.

Photophysical measurements: UV-Vis spectra were recorded on a HITACHI U-3900 spectrophotometer. The steady-state emission spectra and lifetime studies were measured with Edinburgh FL 900 photon-counting system. Both wavelength-dependent excitation and emission responses of the fluorimeter were calibrated. Spectral grade solvents (Merck) were used as received. To determine the photoluminescence quantum yield in solution, samples were degassed using at least three freeze-pump-thaw cycles. The solution quantum yields are calculated using the standard sample which has a known quantum yield, according to the following equation

$$\Phi = \Phi_R \frac{I}{I_R} \frac{A_R}{A} \frac{\eta^2}{\eta_R^2} \quad (1)$$

where Φ is the quantum yield, the subscript R refers to the reference compound of known quantum yield, I is the integrated fluorescence intensity and η is the refractive index of the solvent. A is the absorbance at the excitation wavelength with the value of absorbance between 0.02 ~ 0.05.

Lifetime studies were performed by an Edinburgh FL 900 time correlated single photon counting (TCSPC) system with an EPL-375 diode laser as the excitation source. Data were analyzed using a nonlinear least-squares procedure in combination with an iterative convolution method. The emission decays were fitted by the sum of exponential functions with a temporal resolution of ~ 300 ps after the deconvolution of instrument response function.

Photodegradation studies. These complexes were dissolved in deaerated toluene with conc. of 5 x 10^{-5} $_{M}$ and the solution was transferred to UV cuvettes equipped with Teflon stopcock. These cuvettes were degassed via three freeze-pump-thaw cycles and the cuvettes were filled with argon and the upper portion of stopcock were covered with aluminum foil to prevent the radiation damage. All solutions were irradiated with simulated solar irradiation at 620 W/m^2 and 35 °C using Atlas Suntest CPS+ Xenon Test Instrument, for which the experimental setup is depicted below:

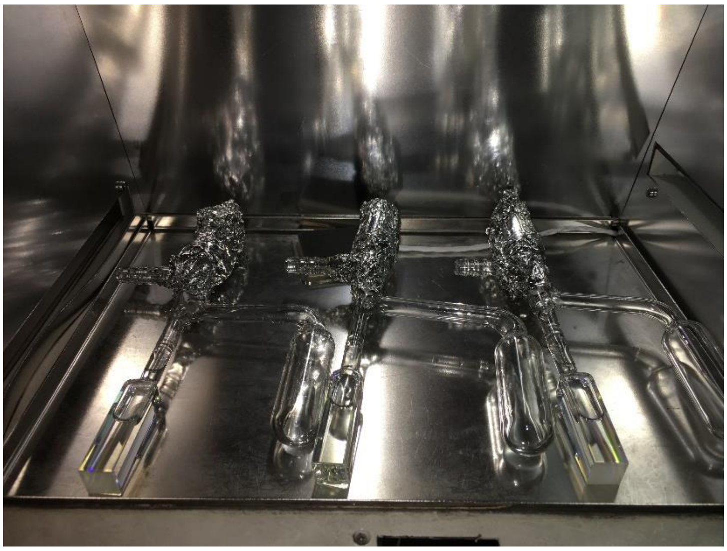

The irradiation intervals were chosen individually for each sample solution. After each irradiation cycle, the samples were analyzed using Edinburgh fluorometer (FLS920). The remaining emitter concentration was determined from their emission intensity under constant excitation power.

Computational Method: All calculations were performed with the Gaussian 09 program package.[4] The geometry optimization of ground states of the three Ir(III) complexes are simulated with density functional theory (DFT) at the B3LYP/LANL2DZ (Ir) and B3LYP/6-31g(d,p) (H, C, N, F, Cl) levels using CH_2Cl_2 as the as the solvent. The optimized structures of the three Ir(III) complexes are then used to calculate the five lowest singlet ($S_0 \rightarrow S_5$) and triplet optical electronic transitions ($S_0 \rightarrow T_5$) using the time–dependent density functional theory (TD–DFT) method. The solvent effect is based on the polarizable continuum model (PCM), which is supported implemented in the Gaussian 09 program. For both singlet and triplet optical transitions Mulliken population analysis (MPA) is applied to obtain the electron density distribution of each atom in specific molecular orbital of the Ir(III) complexes as well as to calculate the metal-to-ligand charge transfer (MLCT) in each assignment during the singlet and triplet optical transitions. The triplet and singlet excited state S_1 and T_1 were also optimized. The electronic configurations of ^{3}MC *dd* and ^{3}MLCT states were calculated following the methodology in the literature.[5] The ^{3}MLCT state geometry was obtained by performing geometry optimization along the triplet ground state potential energy surface (PES), using the core arrangement derived from the optimized structure of the ground state as the initial geometry. As for the ^{3}MC *dd* state, because the electron density distributions of HOMO are mainly localized on the central Ir atom, the calculation was starting with a distorted geometry,

for which the metal-ligand bondings are largely elongated (around ~3Å), such that its associated energy is expected to be far away from the global minimum along the PES, and then the geometry optimization is performed. Accordingly, the optimization is able to fall into the presumably shallow local minimum associated with the ^{3}MC *dd* excited state.

Device fabrication and thin film PL measurement. Before device fabrication, pre-cleaned ITO-coated glass substrates with a sheet resistance of 15 Ω square^{-1} were subjected to UV-ozone treatment for 20 min. All the chemicals are purchased from Lumtec (Taiwan) and organic films were deposited at the rate of 0.5 ~ 1.0 Å s^{-1} by thermal evaporation in a deposition chamber with a base vacuum of 1×10^{-6} Torr. *J*-*V* (current density-voltage) characteristics were recorded with a Keithley 2400 Sourcemeter. Electroluminescence spectra and $CIE_{x,y}$ color coordinates were measured with a Spectrascan PR650 photometer. Time-dependent transition PL (excitation: TCSPC 375 nm) and absolute PLQY (excitation: Xe lamp 360 nm) of doped films (10 *wt*% in bis[2-(diphenylphosphino)phenyl]ether oxide, (DPEPO)) were recorded using an Edinburgh Instruments FLS980 spectrophotometer.

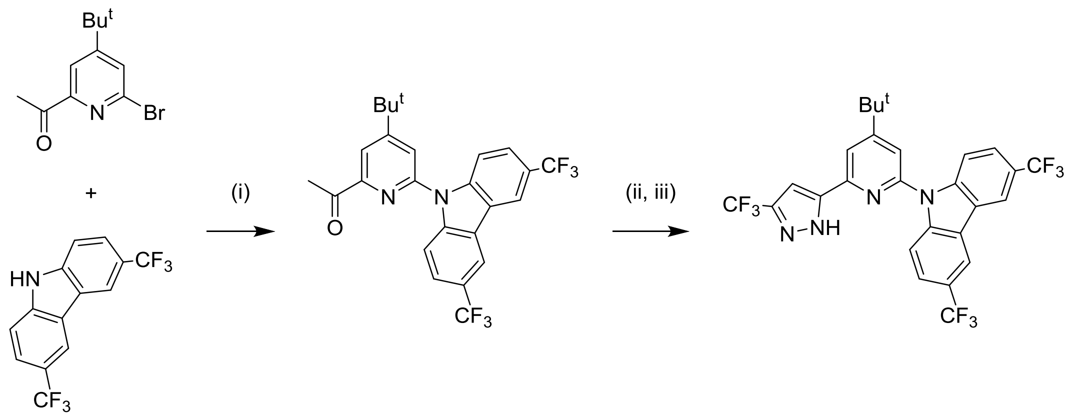

Scheme S1. The synthetic route to [(pzy^{tB}Cz^{F6})H_2] followed conditions : (i) CuI, K_3PO_4, 1,2,diaminocyclohexane, 1,4-dioxane, (ii) NaOEt, CF_3CO_2Et, THF, reflux; (iii) N_2H_4, EtOH, reflux.

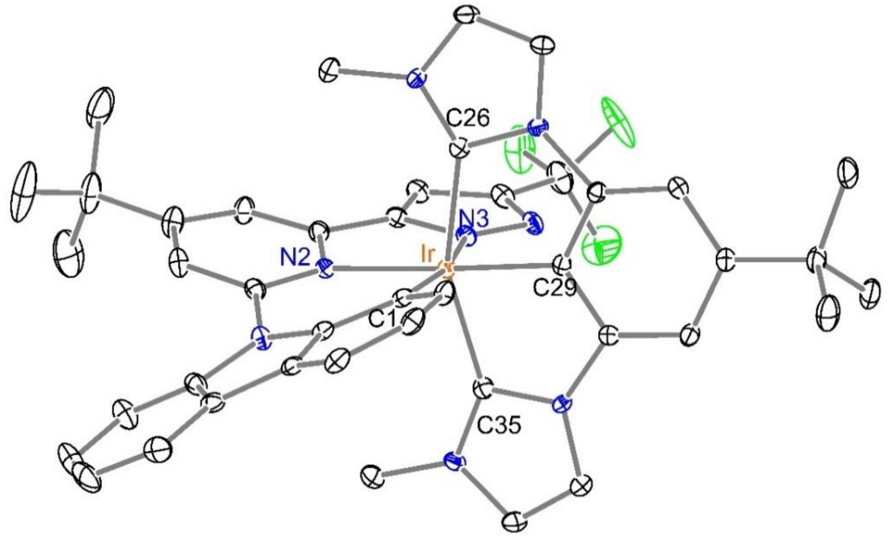

Figure S1. Structural drawing of **Cz-0** with thermal ellipsoids shown at 30% probability level. Selected bond distances: Ir-C(29) = 1.975(3), Ir-C(1) = 2.034(3), Ir-C(35) = 2.046(3), Ir-C(26) = 2.059(3), Ir-N(3) = 2.104(3) and Ir-N(2) = 2.144(2). Selected bond angles: C(26)-Ir-C(35) = 155.30(11), C(29)-Ir-N(2) = 175.18(11) and C(1)-Ir-N(3) = 168.14(10)°.

Table S1(a). The calculated wavelengths, transition probabilities and charge transfer character of the optical absorptions for Ir(III) metal complex **Cz-1** in CH_2Cl_2.

State	λ [nm]	*f*	Assignments	MLCT
T_1	421.7	0	HOMO→LUMO(43%) HOMO-2→LUMO+1(14%) HOMO-3→LUMO+1(9%) HOMO-3→LUMO(8%) HOMO→LUMO+1(6%)	21.60%
T_2	406.3	0	HOMO→LUMO(21%) HOMO-3→LUMO+1(19%) HOMO-3→LUMO(15%) HOMO-2→LUMO+1(11%) HOMO→LUMO+2(7%)	17.94%
T_3	395.7	0	HOMO→LUMO(14%) HOMO-4→LUMO+2(14%) HOMO→LUMO+2(12%) HOMO-2→LUMO(11%) HOMO-4→LUMO(7%) HOMO→LUMO+4(7%) HOMO-5→LUMO+2(5%)	13.86%
T_4	386.5	0	HOMO-2→LUMO(49%) HOMO→LUMO(11%) HOMO-1→LUMO(11%) HOMO→LUMO+1(11%)	19.11%
S_1	384.9	0.0405	HOMO→LUMO(97%)	29.94%
T_5	380.1	0	HOMO-1→LUMO(26%) HOMO-1→LUMO+5(23%) HOMO→LUMO+3(10%) HOMO-5→LUMO+3(6%) HOMO-1→LUMO+7(6%)	16.17%
S_2	370.3	0.0076	HOMO-1→LUMO(97%)	27.19%
S_3	353.9	0.0927	HOMO→LUMO+1(72%) HOMO-2→LUMO(23%)	28.28%
S_4	347.5	0.0813	HOMO-2→LUMO(70%) HOMO→LUMO+1(23%)	20.48%
S_5	336.5	0.0012	HOMO-1→LUMO+1(94%)	28.76%

Table S1(b). The calculated wavelengths, transition probabilities and charge transfer character of the lowest emission for **Cz-1** in CH_2Cl_2.

State	λ [nm]	*f*	Assignments	MLCT
$T_1 \rightarrow S_0$	527.1	0	LUMO→HOMO(76%) LUMO→HOMO-2(13%)	26.53%
$S_1 \rightarrow S_0$	488.7	0.0311	LUMO→HOMO(99%)	27.28%

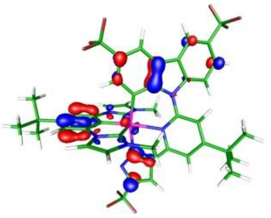<br>HOMO-5 (–6.21 eV)<br>Ir: 4.21%	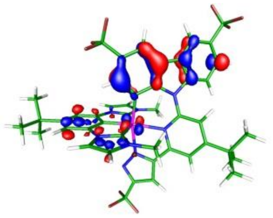<br>HOMO-4 (–6.09 eV)<br>Ir: 6.78%	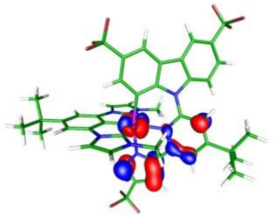<br>HOMO-3 (–5.94 eV)<br>Ir: 21.59%
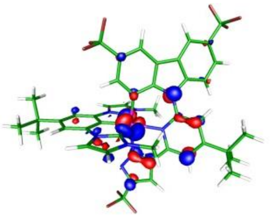<br>HOMO-2 (–5.64 eV)<br>Ir: 21.19%	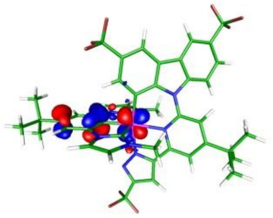<br>HOMO-1 (–5.40 eV)<br>Ir: 30.95%	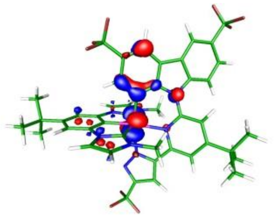<br>HOMO (–5.30 eV)<br>Ir: 33.79%
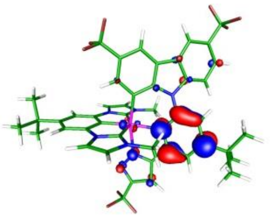<br>LUMO (–2.08 eV)<br>Ir: 2.92%	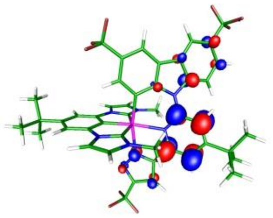<br>LUMO+1 (–1.15 eV)<br>Ir: 0.35%	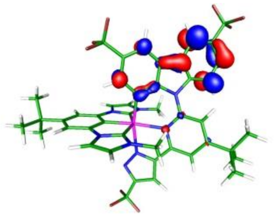<br>LUMO+2 (–1.00 eV)<br>Ir: 0.56%
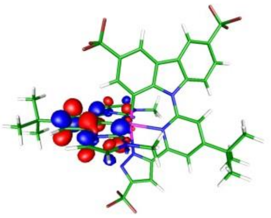<br>LUMO+3 (–0.53 eV)<br>Ir: 1.88%	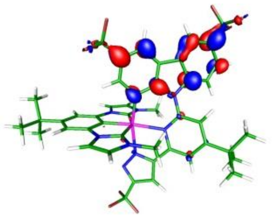<br>LUMO+4 (–0.45 eV)<br>Ir: 2.16%	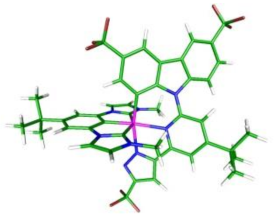<br>optimized structure

Figure S2(a). Frontier molecular orbitals for the ground state S_0 of Ir(III) metal complex **Cz-1**. The electron density distributions of Ir atom in each molecular orbital are showed. For the clarity of viewing, the optimized structure with no involvement of frontier orbitals is shown at the last figure.

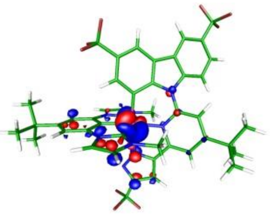	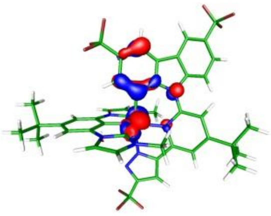	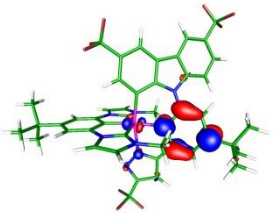
HOMO-2 (–5.62 eV)<br>Ir: 37.69%	HOMO (–5.13 eV)<br>Ir: 33.58%	LUMO (–1.74 eV)<br>Ir: 4.37%

Figure S2(b). Frontier molecular orbitals for the excited state T_1 of **Cz-1**. The electron density distributions of Ir atom in each molecular orbital are showed. For the clarity of viewing, the optimized structure with no involvement of frontier orbitals is shown at the last figure.

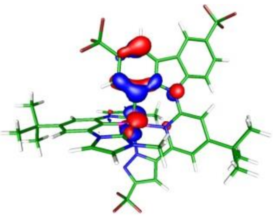	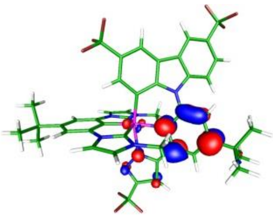	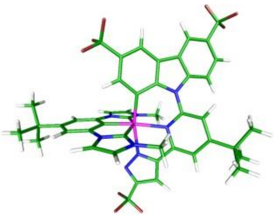
HOMO (–5.01 eV)<br>Ir: 30.96%	LUMO (–1.78 eV)<br>Ir: 3.40%	optimized structure

Figure S2(c). Frontier molecular orbitals for the excited state S_1 of **Cz-1**. The electron density distributions of Ir atom in each molecular orbital are showed. For the clarity of viewing, the optimized structure with no involvement of frontier orbitals is shown at the last figure.

Table S2(a). The calculated wavelengths, transition probabilities and charge transfer character of the optical absorptions for Ir(III) metal complex **Cz-2** in CH_2Cl_2.

State	λ [nm]	*f*	Assignments	MLCT
T_1	417.5	0	HOMO→LUMO(33%) HOMO-1→LUMO+1(13%)<br>HOMO-3→LUMO(11%) HOMO→LUMO+1(10%)<br>HOMO-2→LUMO+1(8%) HOMO-3→LUMO+1(7%)	22.38%
T_2	405.3	0	HOMO→LUMO(28%) HOMO-3→LUMO+1(12%)<br>HOMO-3→LUMO(11%) HOMO-1→LUMO+1(7%)<br>HOMO→LUMO+2(7%) HOMO-4→LUMO+2(5%)	17.59%
T_3	394.3	0	HOMO-4→LUMO+2(18%) HOMO→LUMO(14%)<br>HOMO-1→LUMO(12%) HOMO→LUMO+2(11%)<br>HOMO-4→LUMO(9%) HOMO→LUMO+5(8%)<br>HOMO-4→LUMO+1(5%)	13.31%
T_4	382.2	0	HOMO-1→LUMO(37%) HOMO→LUMO(18%)<br>HOMO-2→LUMO(13%) HOMO→LUMO+1(12%)	21.09%
S_1	377.4	0.0552	HOMO→LUMO(98%)	28.09%
T_5	377.1	0	HOMO-2→LUMO+4(33%)<br>HOMO-1→LUMO+4(19%)<br>HOMO-5→LUMO+3(10%) HOMO→LUMO+3(9%)	11.98%
S_2	349.8	0.1369	HOMO-1→LUMO(67%) HOMO→LUMO+1(27%)	24.70%
S_3	344.2	0.024	HOMO→LUMO+1(62%) HOMO-1→LUMO(28%)<br>HOMO-2→LUMO(6%)	27.59%
S_4	338.9	0.0355	HOMO-2→LUMO(88%) HOMO→LUMO+1(8%)	24.12%
S_5	325.8	0.0399	HOMO→LUMO+2(89%)	27.50%

Table S2(b). The calculated wavelengths, transition probabilities and charge transfer character of the lowest emission for **Cz-2** in CH_2Cl_2.

State	λ [nm]	*f*	Assignments	MLCT
$T_1 \rightarrow S_0$	513.3	0	LUMO→HOMO(65%) LUMO→HOMO-1(12%) LUMO→HOMO-3(11%)	20.33%
$S_1 \rightarrow S_0$	480.7	0.0374	LUMO→HOMO(99%)	24.84%

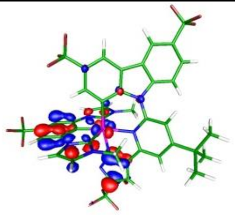<br>HOMO-5 (–6.58 eV)<br>Ir: 6.97%	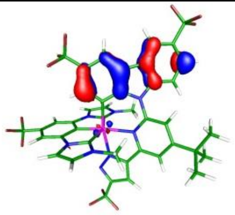<br>HOMO-4 (–6.17 eV)<br>Ir: 3.33%	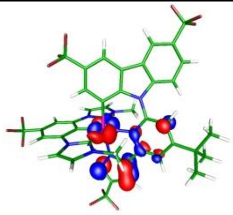<br>HOMO-3 (–6.05 eV)<br>Ir: 24.96%
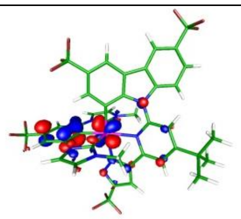<br>HOMO-2 (–5.80 eV)<br>Ir: 27.41%	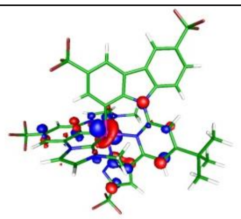<br>HOMO-1 (–5.72 eV)<br>Ir: 27.14%	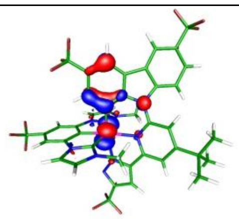<br>HOMO (–5.42 eV)<br>Ir: 31.49%
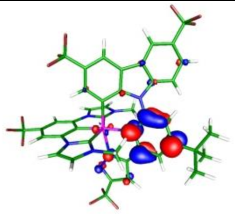<br>LUMO (–2.13 eV)<br>Ir: 2.83%	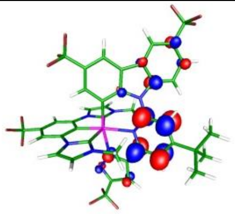<br>LUMO+1 (–1.20 eV)<br>Ir: 0.35%	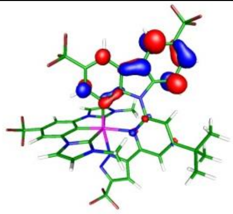<br>LUMO+2 (–1.05 eV)<br>Ir: 0.59%
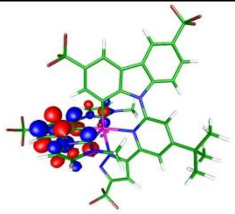<br>LUMO+3 (–0.78 eV)<br>Ir: 1.64%	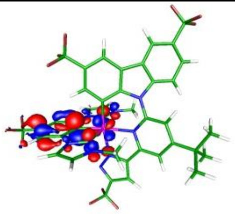<br>LUMO+4 (–0.69 eV)<br>Ir: 10.46%	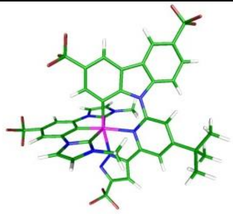<br>optimized structure

Figure S3(a). Frontier molecular orbitals for the ground state S_0 of Ir(III) metal complex **Cz-2**. The electron density distributions of Ir atom in each molecular orbital are showed. For the clarity of viewing, the optimized structure with no involvement of frontier orbitals is shown at the last figure.

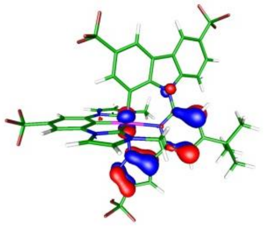<br>HOMO-1 (–5.61 eV)<br>Ir: 12.29%	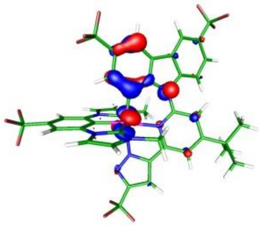<br>HOMO (–5.44 eV)<br>Ir: 25.82%	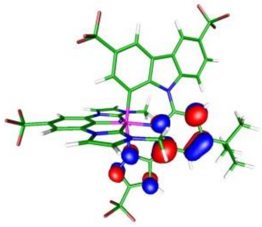<br>LUMO (–1.68 eV)<br>Ir: 1.92%

Figure S3(b). Frontier molecular orbitals for the excited state T_1 of **Cz-2**. The electron density distributions of Ir atom in each molecular orbital are showed. For the clarity of viewing, the optimized structure with no involvement of frontier orbitals is shown at the last figure.

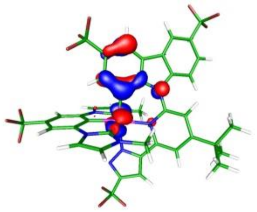<br>HOMO (–5.11 eV)<br>Ir: 28.42%	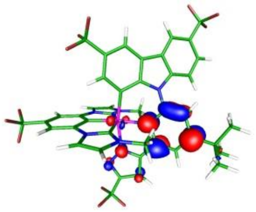<br>LUMO (–1.85 eV)<br>Ir: 3.33%	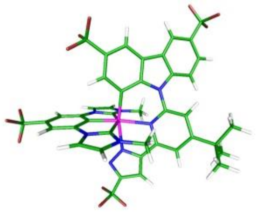<br>optimized structure

Figure S3(c). Frontier molecular orbitals for the excited state S_1 of **Cz-2**. The electron density distributions of Ir atom in each molecular orbital are showed. For the clarity of viewing, the optimized structure with no involvement of frontier orbitals is shown at the last figure.

Table S3(a). The calculated wavelengths, transition probabilities and charge transfer character of the optical absorptions for Ir(III) metal complex **Cz-3** in CH_2Cl_2.

State	λ [nm]	*f*	Assignments	MLCT
T_1	418.5	0	HOMO→LUMO(34%) HOMO-1→LUMO+1(11%) HOMO-3→LUMO(11%) HOMO→LUMO+1(9%) HOMO-2→LUMO+1(9%) HOMO-3→LUMO+1(7%)	22.29%
T_2	406.2	0	HOMO→LUMO(29%) HOMO-3→LUMO+1(13%) HOMO-3→LUMO(11%) HOMO-1→LUMO+1(6%) HOMO→LUMO+2(6%) HOMO-2→LUMO+1(5%)	18.81%
T_3	395	0	HOMO-4→LUMO+2(18%) HOMO→LUMO(13%) HOMO→LUMO+2(12%) HOMO-1→LUMO(11%) HOMO-4→LUMO(9%) HOMO→LUMO+5(8%) HOMO-2→LUMO(5%) HOMO-4→LUMO+1(5%)	14.98%
T_4	384.1	0	HOMO-1→LUMO(36%) HOMO→LUMO(18%) HOMO-2→LUMO(16%) HOMO→LUMO+1(12%)	22.14%
S_1	379.4	0.0497	HOMO→LUMO(98%)	28.77%
T_5	376.9	0	HOMO-2→LUMO+4(31%) HOMO-1→LUMO+4(22%) HOMO-5→LUMO+3(10%) HOMO→LUMO+3(9%)	12.45%
S_2	351.5	0.1251	HOMO-1→LUMO(65%) HOMO→LUMO+1(28%)	25.55%
S_3	346.3	0.0205	HOMO→LUMO+1(58%) HOMO-1→LUMO(31%) HOMO-2→LUMO(8%)	28.32%
S_4	341.1	0.0397	HOMO-2→LUMO(85%) HOMO→LUMO+1(11%)	23.80%
S_5	326.5	0.0381	HOMO→LUMO+2(89%)	28.18%

Table S3(b). The calculated wavelengths, transition probabilities and charge transfer character of the lowest emission for **Cz-3** in CH_2Cl_2.

State	λ [nm]	*f*	Assignments	MLCT
$T_1 \rightarrow S_0$	510	0	LUMO→HOMO(79%) LUMO→HOMO-1(9%)	24.48%
$S_1 \rightarrow S_0$	483	0.0346	LUMO→HOMO(99%)	25.46%

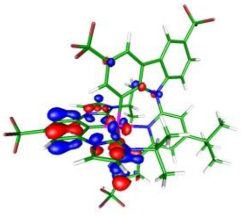<br>HOMO-5 (–6.38 eV)<br>Ir: 6.68%	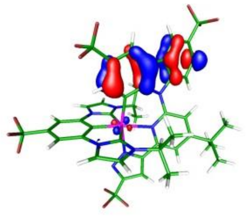<br>HOMO-4 (–6.17 eV)<br>Ir: 3.86%	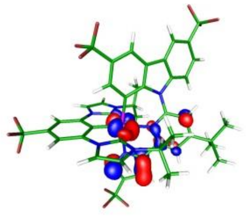<br>HOMO-3 (–6.05 eV)<br>Ir: 23.93%
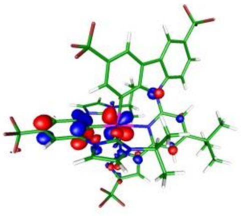<br>HOMO-2 (–5.78 eV)<br>Ir: 26.71%	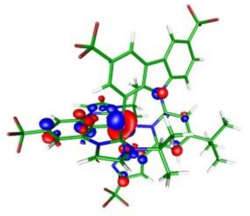<br>HOMO-1 (–5.71 eV)<br>Ir: 28.42%	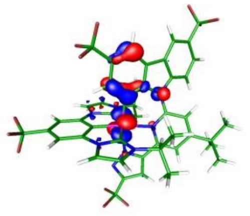<br>HOMO (–5.42 eV)<br>Ir: 32.20%
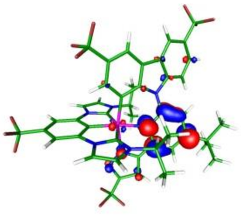<br>LUMO (–2.15 eV)<br>Ir: 2.84%	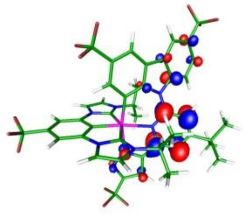<br>LUMO+1 (–1.21 eV)<br>Ir: 0.33%	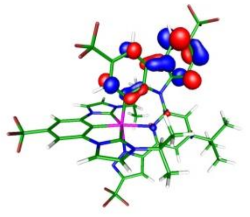<br>LUMO+2 (–1.05 eV)<br>Ir: 0.54%
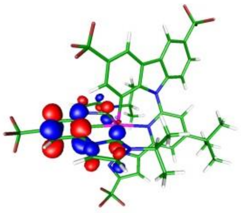<br>LUMO+3 (–0.76 eV)<br>Ir: 1.52%	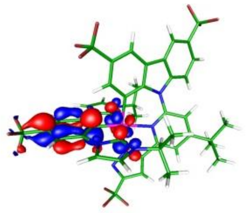<br>LUMO+4 (–0.67 eV)<br>Ir: 10.12%	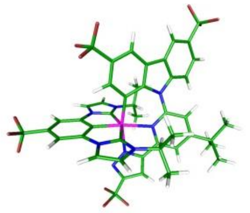<br>optimized structure

Figure S4(a). Frontier molecular orbitals for the ground state S_0 of Ir(III) metal complex **Cz-3**. The electron density distributions of Ir atom in each molecular orbital are showed. For the clarity of viewing, the optimized structure with no involvement of frontier orbitals is shown at the last figure.

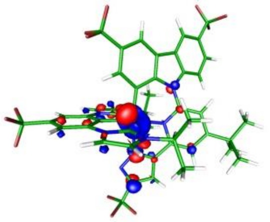	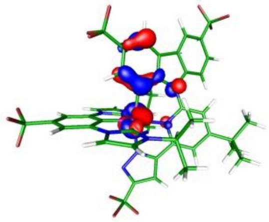	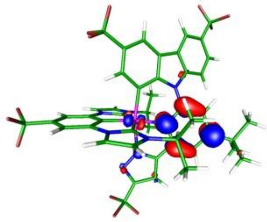
HOMO-1 (–5.74 eV)	HOMO (–5.23 eV)	LUMO (–1.80 eV)
Ir: 35.62%	Ir: 31.46%	Ir: 4.07%

Figure S4(b). Frontier molecular orbitals for the excited state T_1 of **Cz-3**. The electron density distributions of Ir atom in each molecular orbital are showed. For the clarity of viewing, the optimized structure with no involvement of frontier orbitals is shown at the last figure.

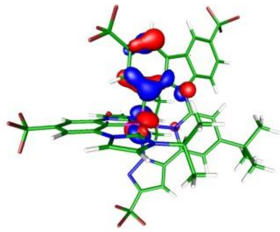	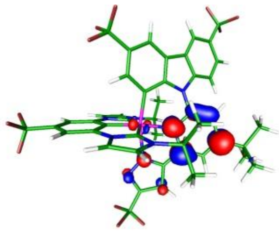	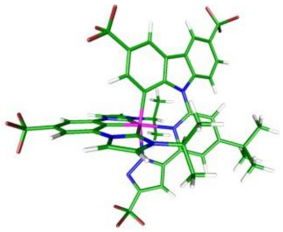
HOMO (–5.09 eV)	LUMO (–1.84 eV)	optimized structure
Ir: 29.05%	Ir: 3.33%	

Figure S4(c). Frontier molecular orbitals for the excited state S_1 of **Cz-3**. The electron density distributions of Ir atom in each molecular orbital are showed. For the clarity of viewing, the optimized structure with no involvement of frontier orbitals is shown at the last figure.

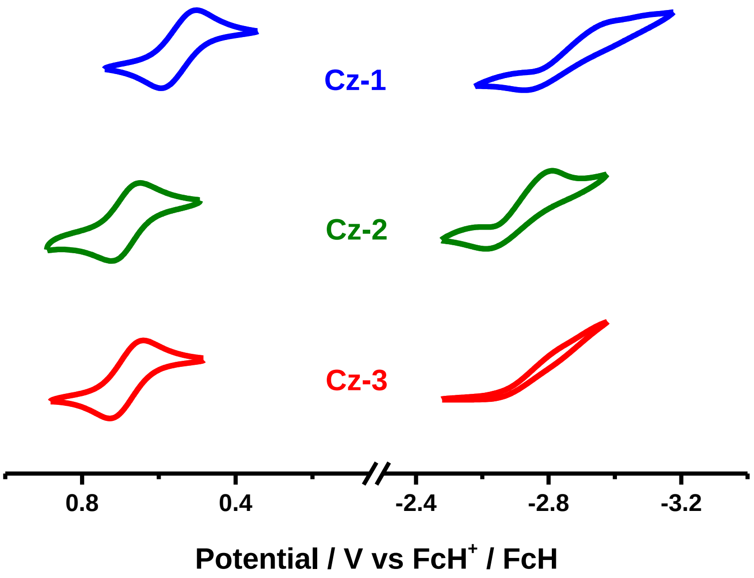

Figure S5. Cyclic voltammetry measurement of the Ir(III) phosphors **Cz-1** – **Cz-3** for this work.

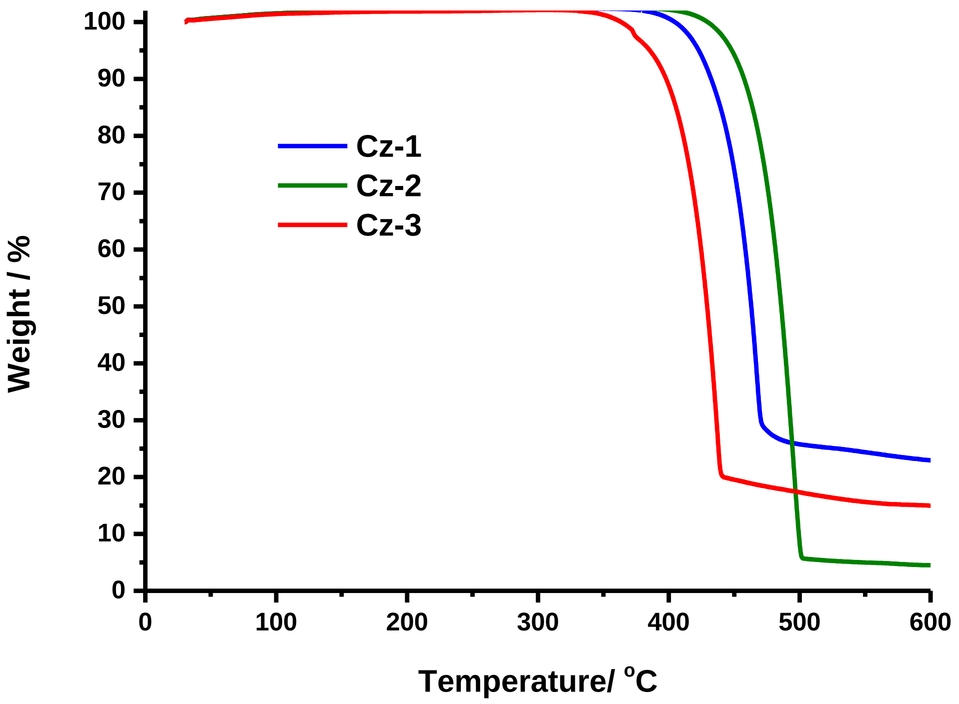

Figure S6. The thermal property for Ir(III) complexes **Cz-1** – **Cz-3** of this study.

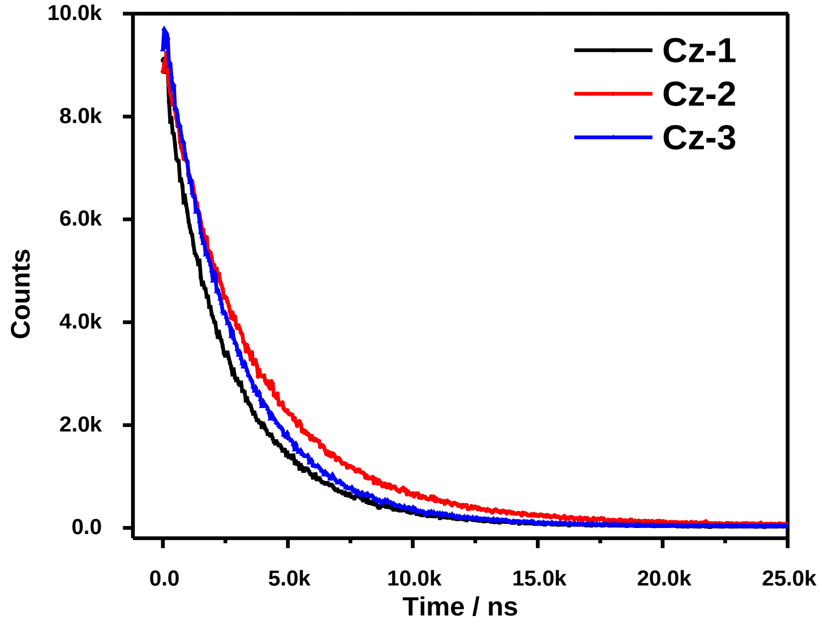

Figure S7.Time-dependent PL measurement of emitters **Cz-1** - **Cz-3** co-deposited in DPEPO film with conc. of 10 *wt*%.

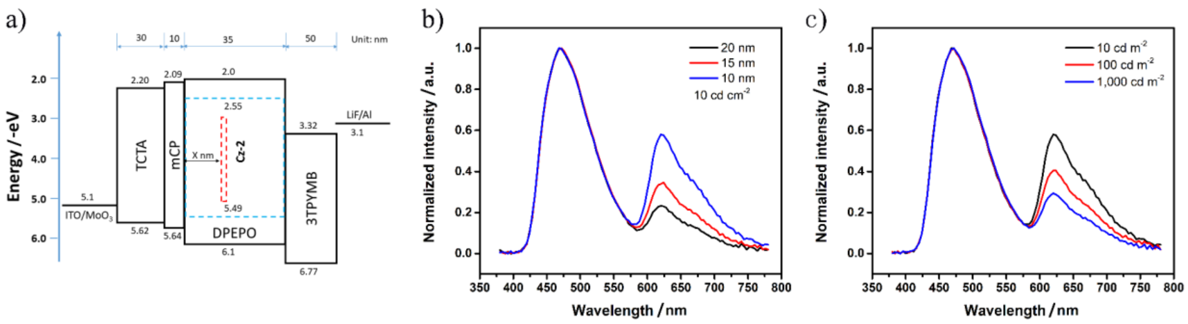

Figure S8. a) Scheme of device with a sensing layer (represented by red rectangle) device configuration :ITO/MoO_3 (1 nm)/TCTA (30 nm)/mCP (10 nm)/DPEPO: 10 wt% of **Cz-2** (35 nm)/3TPYMB (50 nm)/LiF (1 nm)/Al (120 nm), [Ir(piq)$_2$acac] is co-doped at 2 wt% and a width of 2 nm and at distance (x = 10, 15 and 20 nm) to the EML/mCP interface in the EMLs, b) EL spectra of the devices with different x, c) EL spectra at different luminance with x = 10 nm.

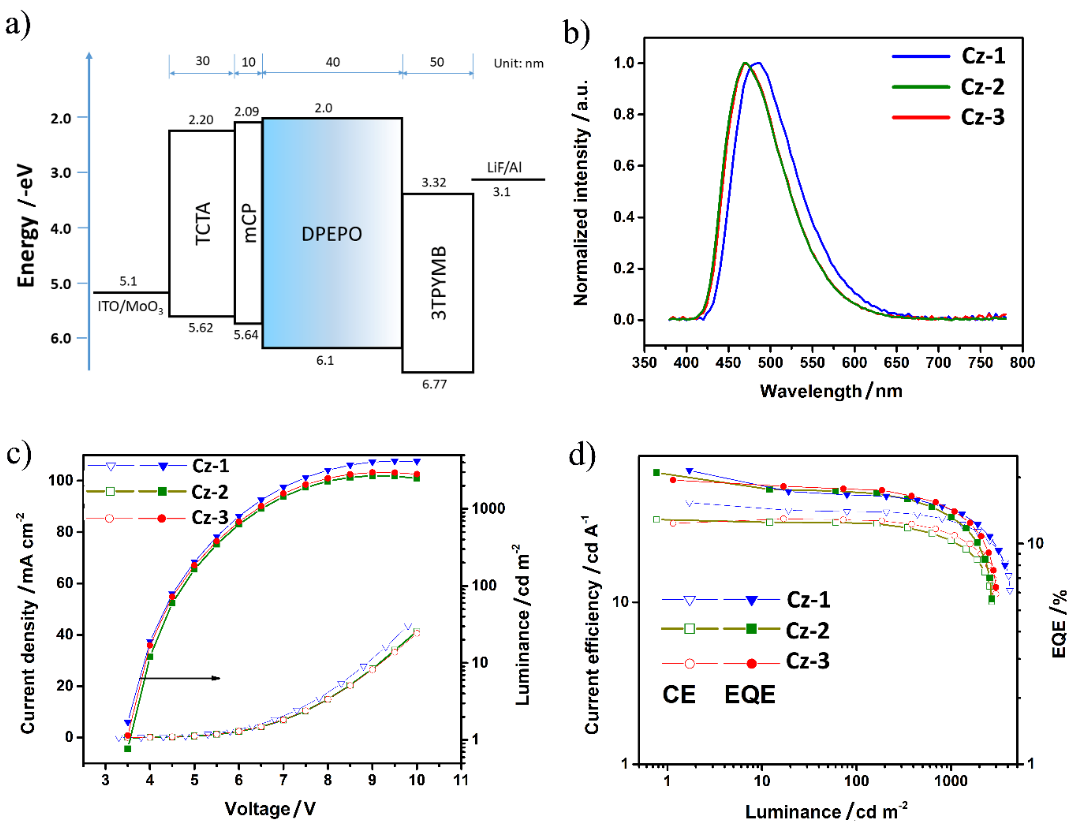

Figure S9. a) Gradient doping device: ITO/MoO_3 (1 nm)/TCTA (30 nm)/mCP (10 nm)/DPEPO: 15-6 *wt*% dopant (40 nm)/ 3TPYMB (50 nm)/LiF (1 nm)/Al (120 nm), doping concentration is linearly decreasing from 15 *wt*% at the EML/mCP interface to 6 *wt*% at the EML/3TPYMB interface (shown by gradually fading blue color in EML), b) EL spectra of the device, c) Current density-voltage-luminescence characteristics, and d) plots of current efficiency-luminance-EQE.

References

[1] V. C. Vargas, R. J. Rubio, T. K. Hollis, M. E. Salcido, *Org. Lett.* **2003**, *5*, 4847-4849.

[2] D. Volz, D. M. Zink, T. Bocksrocker, J. Friedrichs, M. Nieger, T. Baumann, U. Lemmer, S. Bräse, *Chem. Mater.* **2013**, *25*, 3414-3426.

[3] C.-C. Chou, P.-H. Chen, F.-C. Hu, Y. Chi, S.-T. Ho, J.-J. Kai, S.-H. Liu, P.-T. Chou, *J. Mater. Chem. A* **2014**, *2*, 5418-5426.

[4] M. J. Frisch, G. W. Trucks, H. B. Schlegel, G. E. Scuseria, M. A. Robb, J. R. Cheeseman, G. Scalmani, V. Barone, B. Mennucci, G. A. Petersson, H. Nakatsuji, M. Caricato, X. Li, H. P. Hratchian, A. F. Izmaylov, J. Bloino, G. Zheng, J. L. Sonnenberg, M. Hada, M. Ehara, K. Toyota, R. Fukuda, J. Hasegawa, M. Ishida, T. Nakajima, Y. Honda, O. Kitao, H. Nakai, T. Vreven, J. A. Montgomery, J. E. Peralta, F. Ogliaro, M. Bearpark, J. J. Heyd, E. Brothers, K. N. Kudin, V. N. Staroverov, R. Kobayashi, J. Normand, K. Raghavachari, A. Rendell, J. C. Burant, S. S. Iyengar, J. Tomasi, M. Cossi, N. Rega, J. M. Millam, M. Klene, J. E. Knox, J. B. Cross, V. Bakken, C. Adamo, J. Jaramillo, R. Gomperts, R. E. Stratmann, O. Yazyev, A. J. Austin, R. Cammi, C. Pomelli, J. W. Ochterski, R. L. Martin, K. Morokuma, V. G. Zakrzewski, G. A. Voth, P. Salvador, J. J. Dannenberg, S. Dapprich, A. D. Daniels, Ö. Farkas, J. B. Foresman, J. V. Ortiz, J. Cioslowski, D. J. Fox, *Gaussian 09, Revision D.01; Gaussian Inc.* **2013**, Wallingford, CT.

[5] M. Abrahamsson, M. J. Lundqvist, H. Wolpher, O. Johansson, L. Eriksson, J. Bergquist, T. Rasmussen, H.-C. Becker, L. Hammarström, P.-O. Norrby, B. Åkermark, P. Persson, *Inorg. Chem.* **2008**, *47*, 3540-3548.
